# *Lycium barbarum* L. Polysaccharide (LBP) Reduces Glucose Uptake via Down-Regulation of SGLT-1 in Caco2 Cell

**DOI:** 10.3390/molecules22020341

**Published:** 2017-02-22

**Authors:** Huizhen Cai, Xiaohui Yang, Qian Cai, Binbin Ren, Hongyan Qiu, Zhiqing Yao

**Affiliations:** 1 Department of Nutrition and Food Hygiene, School of Public Health, Ningxia Medicine University, Yinchuan 750004, Ningxia, China; CHZ.Liu08@gmail.com; 2 Department of Sanitary Chemistry, School of Public Health, Ningxia Medicine University, Yinchuan 750004, Ningxia, China; yangxh@nxmu.edu.cn; 3 Department of Experimental Center, School of Public Health, Ningxia Medicine University, Yinchuan 750004, Ningxia, China; caiqian1212@126.com (Q.C.); renbb@nxmu.edu.cn (B.R.); 4 Department of Epidemiology and Health Statistics, School of Public Health, Ningxia Medicine University, Yinchuan 750004, Ningxia, China; yanzide80@163.com

**Keywords:** *Lycium barbarum* L. polysaccharide, Caco2 cell monolayer, SGLT-1, GLUT-2

## Abstract

*Lycium barbarum* L. polysaccharide (LBP) is prepared from *Lycium barbarum* L. (*L. barbarum*), which is a traditional Chinese medicine. LPB has been shown to have hypoglycemic effects. In order to gain some mechanistic insights on the hypoglycemic effects of LBP, we investigated the uptake of LBP and its effect on glucose absorption in the human intestinal epithelial cell line Caco2 cell. The uptake of LBP through Caco2 cell monolayer was time-dependent and was inhibited by phloridzin, a competitive inhibitor of SGLT-1. LPB decreased the absorption of glucose in Caco2 cell, and down-regulated the expression of SGLT-1. These results suggest that LBP might be transported across the human intestinal epithelium through SGLT-1 and it inhibits glucose uptake via down-regulating SGLT-1.

## 1. Introduction

In a previous study we have found that *Lycium barbarum* L. polysaccharide (LBP), a plant extract of *Lycium barbarum* L. (*L. barbarum*), has hypoglycemic properties in diabetics [1]. However, there is a lack of mechanistic insights into the hypoglycemic effects of LBP. Polysaccharides are macromolecules with complex structures, formed by condensation and dehydration of at least ten monosaccharide molecules. It is generally believed that polysaccharides, like starch, cannot be absorbed directly and they are hydrolysed by amylase and disaccharidases into sugars that can be absorbed by the intestine [2]. However, it has not been shown before whether or not LBP can cross the intestinal epithelium. In the present study, LBP was prepared as described previously [1] and we investigated the uptake of LBP and its effect on glucose absorption by the Caco2 monolayer. Caco2 cells can form confluent monolayers with the functional property of transporting epithelia and are widely used for studying drug transport mechanisms [3,4].

## 2. Results

### 2.1. Characterization of LBP

LBP we prepared was a brown powder composed of neutral sugars (78.23%) and acidic sugars (14.83%). The protein content was <6.92%.

#### 2.1.1. Monosaccharide Analysis of LBP

**Figure 1:**
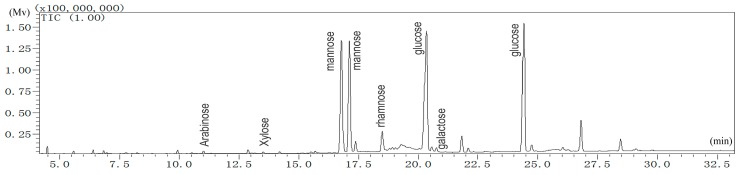
GC-MS analysis of LBP.

The monosaccharide composition of LBP was elucidated based on the GC retention times of sugar standards (Figure 1). The retention time of d-arabinose was 11.010 min. d-xylose was 13.510 min. d-mannose was 16.785 and 17.120 min. l-rhamnose was 18.495 min. d-glucose was 20.365 and 24.435 min. d-galactose was 20.760 min. d-mannose and d-glucose each had two peaks, corresponding to the different configuration of mannose or glucose. The quantified relative content (%) of monosaccharides was as follows: arabinose, 0.31; xylose, 0.16; mannose, 41.68; rhamnose, 3.46; glucose, 53.83; galactose, 0.56. This result clearly demonstrated that mannose and glucose were the dominant monosaccharides in LBP. This is quite different from that previously reported for LBP isolated from the same species [5]. These differences may be due to different sources of plant collection, different isolation procedures, or likely from the different methods used to determine the monosaccharide ratio.

#### 2.1.2. Molecular Weights

**Figure 2:**
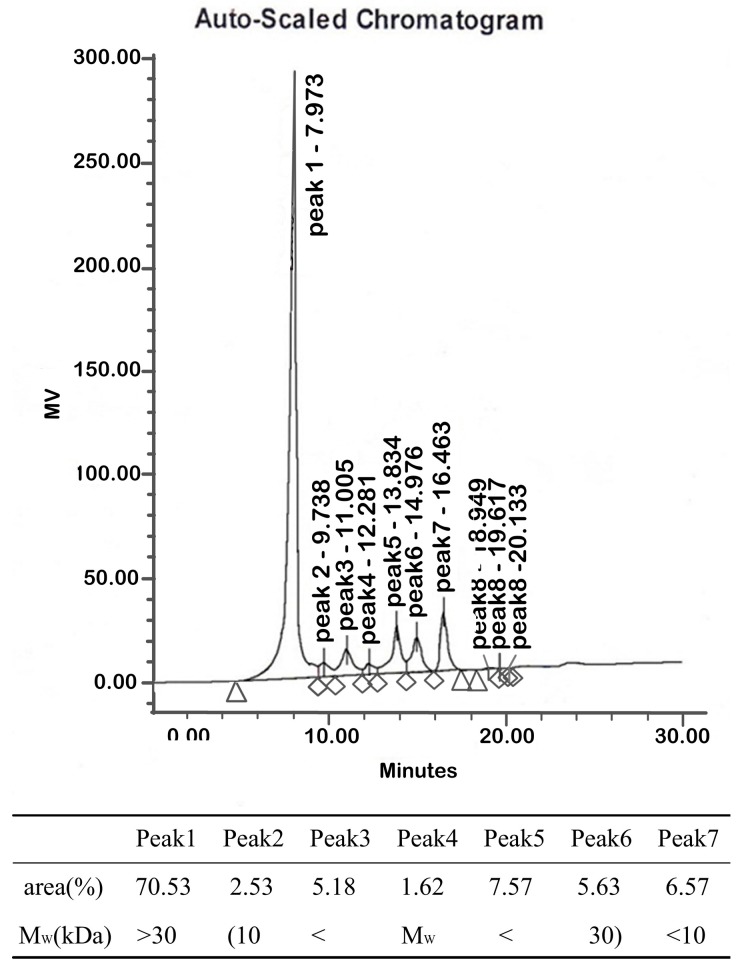
Molecular weight of LBP. Peak 1 has a molecular weight higher than 30 kDa. The molecular weights of peak 2 to peak 6 lie between 10 kDa to 30 kDa. Peak 7 has a molecular weight lower than 10 kDa.

Peak 1 had a molecular weight higher than 30 kDa and an area of 70.53% (Figure 2). The molecular weights of peaks 2 to peak 6 lie between 10 kDa to 30 kDa. The areas of peak 2 to peak 6 amount to 22.53%. Peak 7, with an area of 6.57%, had a molecular weight lower than 10 kDa. Therefore, polysaccharides greater than 10 kDa amounted to 93.06% of the polysaccharide fraction of LBP.

#### 2.1.3. UV Analysis

**Figure 3:**
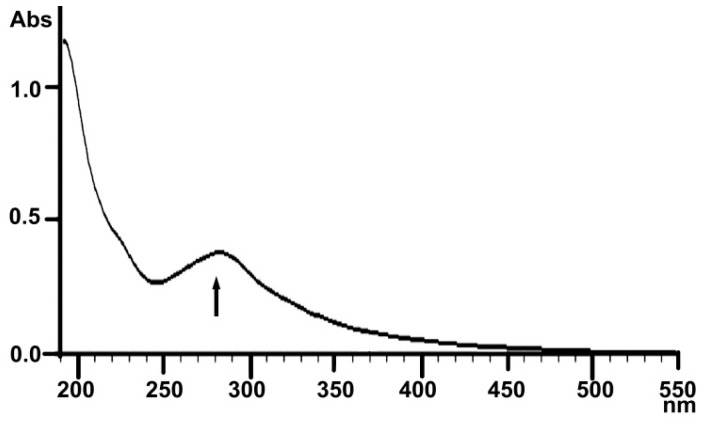
UV spectrum of LBP.

Tyrosine, phenylalanine and tryptophan are common components in proteins. Their contents are similar in various proteins. They show a maximum absorption at 280 nm, so the most common way to determine proteins is by the ultraviolet absorption method. A weak absorbance at 280 nm is shown in the range of 200–550 nm in the UV spectra of LBP (Figure 3), indicating that the LBP contained only a small amount of protein. This finding is similar with previously reported results [6].

#### 2.1.4. Infrared Spectral Analysis

**Figure 4:**
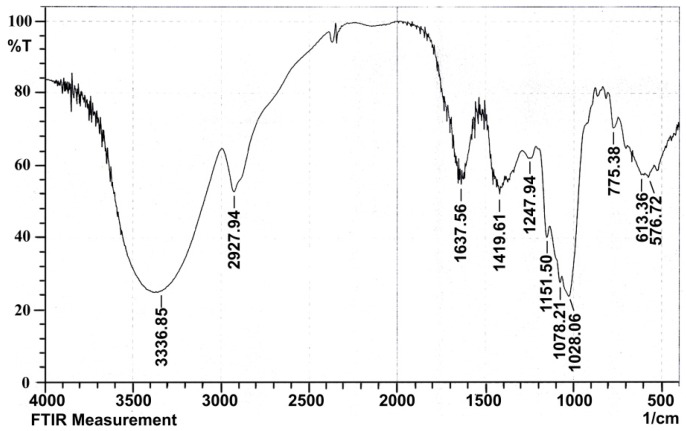
Infrared spectral analysis of LBP.

A typical carbohydrate IR absorption spectrum of LBP is shown in Figure 4. The spectrum exhibited a broadly-stretched intense peak at 3336.85 cm^−1^ for the -OH, -NH_2_ or –NH-group. The moderately strong bands at 2927.94 cm^−1^ indicated the C-H stretching vibration of CH_2_. These are characteristic absorption peaks of polysaccharides. The bands at 1637.56 cm^−1^ and 1419.61 cm^−1^ were attributed to C=O (-NH_2_CONH_3_-) variable angle vibration, proving the presence of uronic acid. The band at 1247.94 cm^−1^ was ascribed to C-O-C stretching vibration of ketone. The absorption at 1151.50 cm^−1^ was due to C-O (-COOH) or C-N stretching vibration. The band at 1078.21 cm^−1^ was assigned to the asymmetric pyranose C-O-C vibration, and the absorption at 1028.06 cm^−1^ was characteristic absorption peak of glycosidic rings, indicating the presence of pyranose moieties. The peak at 921.97 cm^−1^ of LBP was used to infer the position of the rhamnose terminal (-CH_2_-) in the deoxynucleotidyl sugar. The band at 775.38 cm^−1^ was symmetrical stretching vibration of a pyranoid ring.

### 2.2. The Uptake of LBP by Caco2 Cell Monolayer

**Figure 5:**
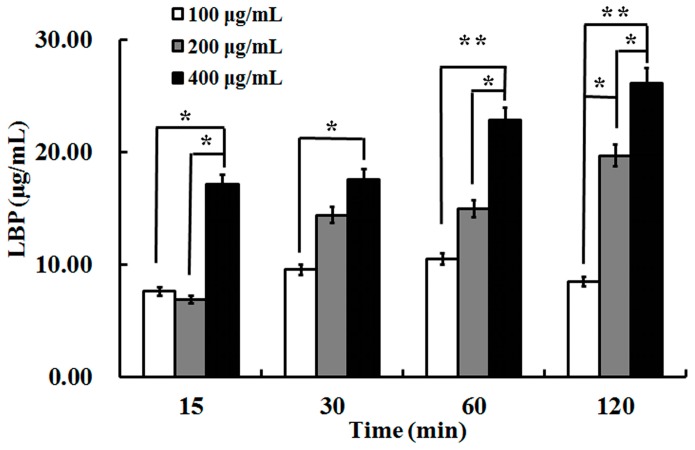
The uptake of LBP at 100, 200, and 400 μg/mL concentrations in Caco2 after 2 h incubation. Values are means ± SD of duplicates, from three experiments. The asterisk indicates a significant difference among samples, * *p* < 0.05; ** *p* < 0.01 (ANOVA followed by SNK-q test).

Uptake of LBP through a Caco2 cell monolayer was examined at various loading concentrations (100, 200, 400 μg/mL, Figure 5). In two-way repeated measures ANOVA, there was an overall difference between groups (*F* = 16.87, *p* < 0.05) and over time (*F* = 6.86, *p* < 0.01), but there was no interaction between the two factors (*F* = 1.84, *p* > 0.05). There was statistical difference between the concentrations of 100 μg/mL and 400 μg/mL at each time, as well as 200 μg/mL with 400 μg/mL at 15, 60 and 120 min. No glucose was detectable in the samples. The results indicated that LBP was uptaken directly by the Caco2 cell monolayer in a time-dependent manner.

### 2.3. Effect of LBP on Glucose

**Figure 6:**
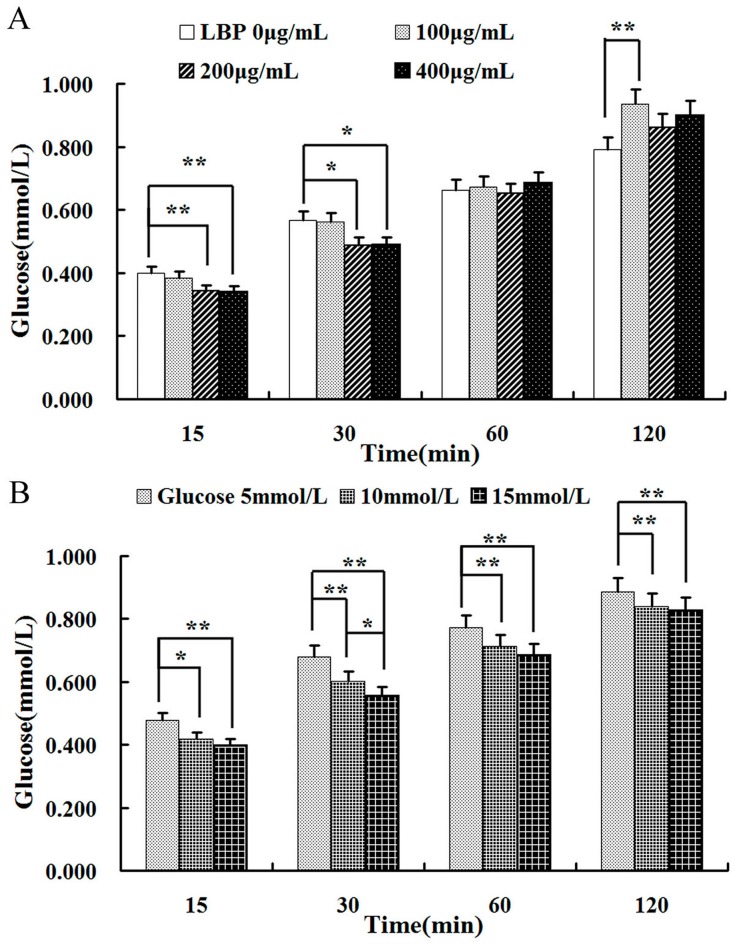
The uptake of LBP on absorption of glucose in Caco2 cells. (**A**) Caco2 cells were treated with glucose (10 mmol/L) and different concentrations of LBP (0, 100, 200 and 400 μg/mL) for 120 min; (**B**) Caco2 cells were treated with LBP (200 μg/mL) and different concentrations of glucose (5, 10 and 15 mmol/L) for 120 min. The data are presented as the mean ± SD (*n* = 3). The asterisk indicate a significant difference among samples, * *p* < 0.05; ** *p* < 0.01 (ANOVA followed by SNK-q test).

Glucose absorption was investigated with different concentrations of LBP (100, 200 and 400 μg/mL, Figure 6A). Glucose (10 mmol/L) absorption decreased significantly in the presence of 200 or 400 μg/mL LBP during the first 30 min (*p* < 0.05 or *p* < 0.01).

However, the inhibitory effect of LBP on glucose absorption faded after 60 min (*p* > 0.05). 100 μg/mL LBP had no effect on glucose absorption during the first 60 min (*p* > 0.05, one-way ANOVA). At 120 min, it promoted the uptake of glucose (*p* < 0.05, one-way ANOVA, post-hoc test). The effects of LBP (at concentrations of 200 μg/mL) were further investigated on the absorption of glucose at different loading concentrations (5, 10 and 15 mmol/L) (Figure 6B). There was an overall difference between groups (*F* = 31.23, *p* < 0.01) and over time (*F* = 1461.17, *p* < 0.01), but there was no interaction of the two factors (*F* = 2.84, *p* > 0.05) on two-way repeated measures ANOVA. There were differences between the concentrations of 5 mmol/L and 10 mmol/L (or 15 mmol/L) at each time point. LBP had better inhibitory effect on higher concentrations of glucose (15 mmol/L).

### 2.4. Involvement of SGLT-1 or GLUT-2 in the Uptake of LBP by Caco2 Monolayer

**Figure 7:**
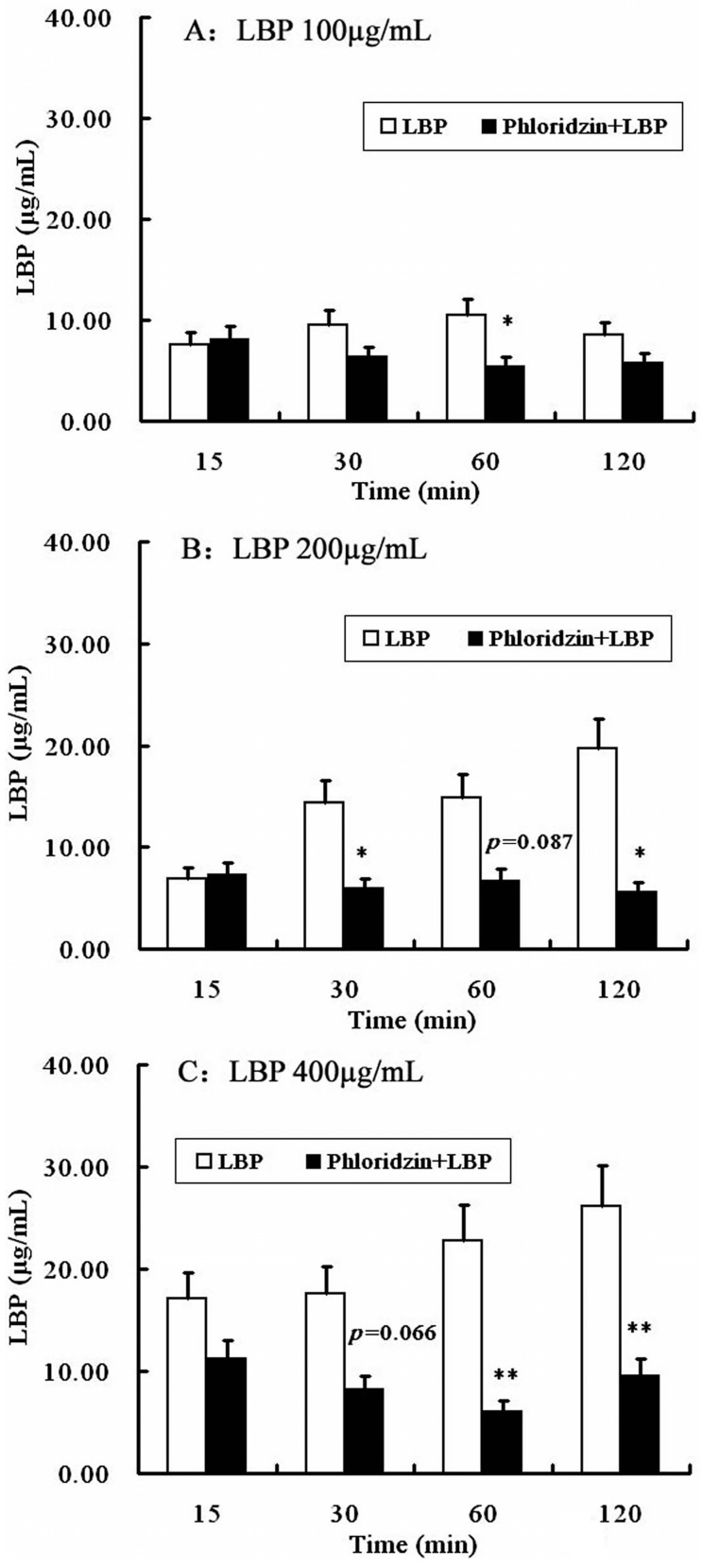
The uptake of LBP after SGLT-1 inhibition in Caco2 cells. (**A**) Caco2 cells were treated with LBP (100 μg/mL) for 120 min after phloridzin (2 mmol/L) pretreating; (**B**) Caco2 cells were treated with LBP (200 μg/mL) for 120 min after phloridzin (2 mmol/L) pretreating; (**C**) Caco2 cells were treated with LBP (400 μg/mL) for 120 min after phloridzin (2 mmol/L) pretreating. The data are presented as the mean ± SD (*n* = 3). The asterisk indicate a significant difference among samples, * *p* < 0.05; ** *p* < 0.01 (Student’s paired *t*-test).

**Figure 8:**
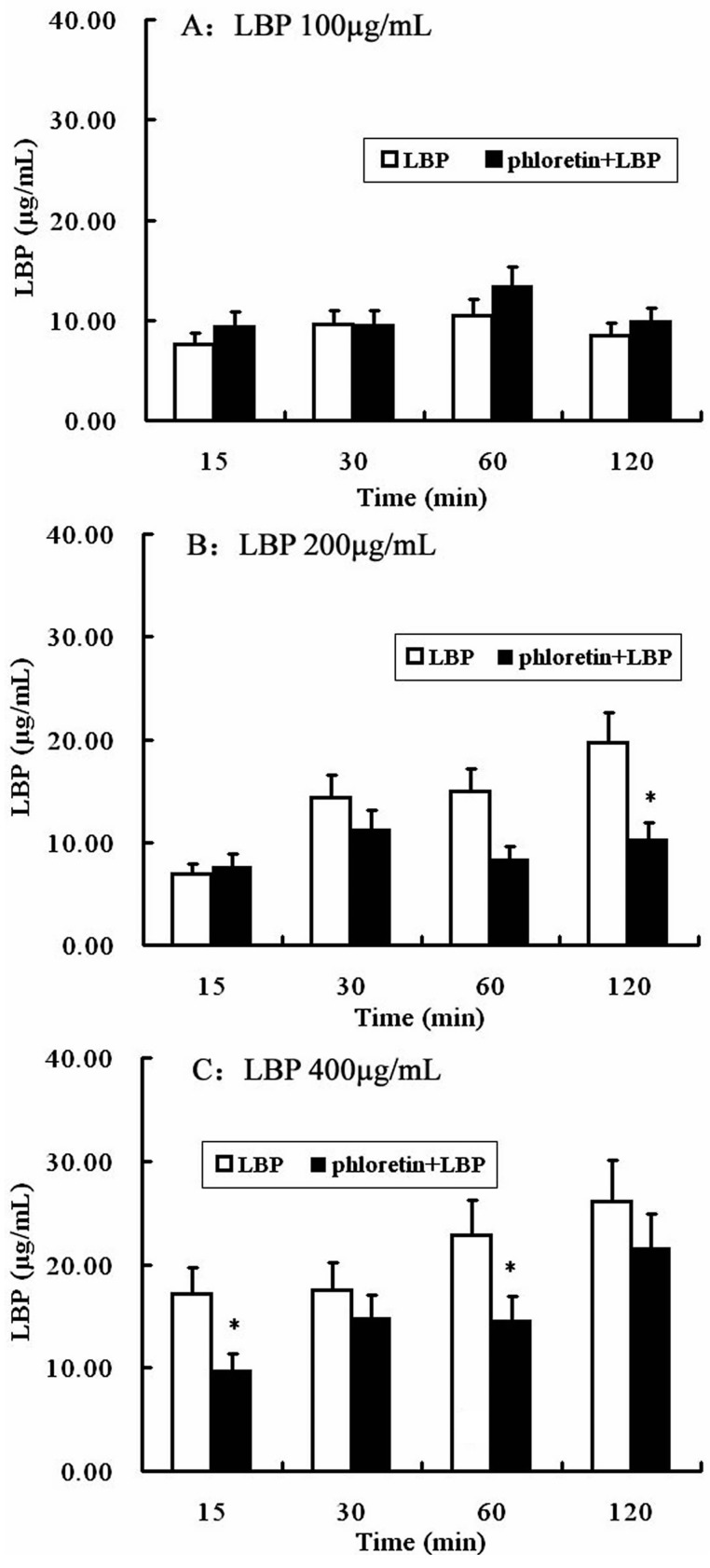
The uptake of LBP after GLUT-1 inhibition in Caco2 cells. (**A**) Caco2 cells were treated with LBP (100 μg/mL) for 120 min after phloretin (2 mmol/L) pretreating; (**B**) Caco2 cells were treated with LBP (200 μg/mL) for 120 min after phloretin (2 mmol/L) pretreating; (**C**) Caco2 cells were treated with LBP (400 μg/mL) for 120 min after phloretin (2 mmol/L) pretreating. The data are presented as the mean ± SD (*n* = 3). The asterisk indicate a significant difference among samples, * *p* < 0.05 (Student’s paired *t*-test).

Then the possible involvement of SGLT-1 or GLUT-2 in the uptake of LBP was studied by pretreating Caco2 monolayer with phloridzin, a SGLT-1 inhibitor, or phloretin, a GLUT-2 inhibitor. As shown in Figure 7, phloridzin (2 mmol/L) pretreatment significantly (*P* < 0.01, two-way repeated measures ANOVA) inhibited the uptake of LBP at different loading concentrations (100, 200 and 400 µg/mL). Pretreatment of the Caco2 monolayer with phloretin (2 mmol/L) did not affect the uptake of LBP at 100 µg/mL, but inhibited the uptake of 200 µg/mL (at 120 min) and 400 µg/mL LBP (at 15 and 60 min) (*P* < 0.05) (Figure 8). 

### 2.5. Effect of LBP on SGLT-1mRNA and GLUT-2mRNA Expression

**Figure 9:**
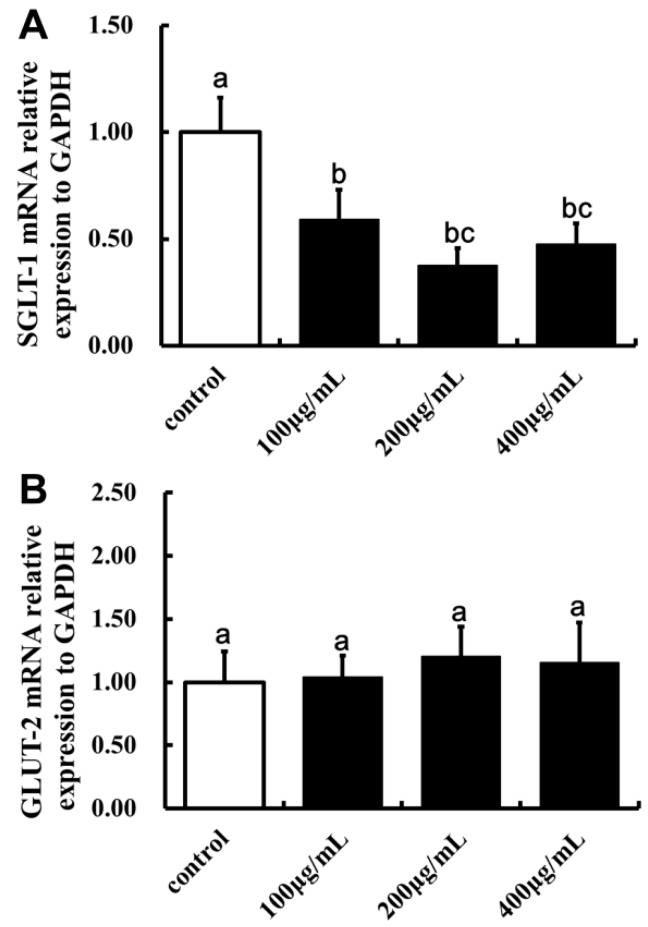
The effect of LBP on the mRNA expression of SGLT-1 and GLUT-2 in Caco2 cells. (**A**) The effect of LBP on the mRNA expression of SGLT-1 in Caco2 cells (one-way ANOVA); (**B**) The effect of LBP on the mRNA expression of GLUT-2 in Caco2 cells (one-way ANOVA). The data are presented as the mean ± SD (*n* = 3). The bars without the same letter differed significantly (*p* < 0.05).

Finally, the effect of LBP on SGLT-1mRNA and GLUT-2 mRNA expression were tested on the Caco2 cells. The expression of SGLT-1 was significantly lower in cells treated with 100, 200 and 400 µg/mL LBP than the control (*p* < 0.05, Figure 9). However, LBP pretreatment did not have a significant effect on the expression of GLUT-2 (*p* > 0.05).

## 3. Discussion

In this study, uptake of LBP and its effect on glucose absorption by the Caco2 monolayer cell was investigated in order to gain mechanistic insights into its previously reported hypoglycemic effects. The Caco2 cell line was originally obtained from a human colon adenocarcinoma and forms confluent monolayer in culture which has been used as a model of the human intestinal epithelium to test drug transport in the gut. Although it is generally believed that polysaccharides like starch cannot be absorbed directly, there have been reports indicating that certain polysaccharides might be transported across the intestinal epithelium. For example, Matsushita et al. [6] showed that Shoyu polysaccharides could be transported across the Caco2 monolayer cell. Garcia-Gonzalez et al. [7] proposed the possibilities of tuning drug loading and release by polysaccharide-based aerogels. They point out that polysaccharide could be used as carriers of poorly water soluble drugs for oral administration. The results show here that LBP could be transported across Caco2 monolayer cell in a time-dependent manner. Our study was consistent with these previous studies and support the notion that an intestinal uptake of certain polysaccharides occurred. However, the products of the polysaccharides still remain a mystery. Humans lack degrading enzymes for certain polysaccharides [8] and have coevolved with a dense consortium of symbiotic distal gut microorganisms in the colon [9]. These microorganisms are adapted to target polysaccharides for their own nutrition. A significant proportion of polysaccharides are fermented by bacterial enzymes and disintegrated into smaller units. Hu et al. [10] showed that the molecular weight (Mw) of polysaccharide from *Plantago asiatica L.* seeds decreased from 1903.1 ± 93.0 kDa to 8.9 ± 0.5 kDa during gastric digestion in vitro, and it continuously decreased as digestion progressed, but there was no monosaccharide released throughout the whole digestion period. In our study, glucose was also not detected during the uptake of LBP. This result supports the view that the polysaccharides might be transported in the form of macromolecules. 

Zhang et al. [11] reported that oat β-glucan was able to inhibit glucose transport, decrease the concentration of available glucose and suppress disaccharide activity in the small intestine. The effect of LBP on glucose absorption showed that glucose absorption was inhibited by LBP in our study. The result indicates that LBP influenced the absorption of glucose and they might have the same transporters. 

The intestine epithelial functions as the first biologic barrier for the ingestion of fats, carbohydrates and proteins. Carbohydrates are degraded into monosaccharides like glucose and maltose and so on. There are three pathways for glucose absorption. The first one is an active transport processes mediated by sodium-dependent glucose cotransporter (SGLT-1) on the intestinal mucosa. The enterocytes glucose transport system is well characterized and expresses SGLT-1 on the apical (brush-border) membrane. The SGLT-1 is responsible for active glucose uptake in enterocytes. The second one is passive diffusion streaming according to concentration gradient mediated by facilitative sodium-independent glucose transporters (GLUT-2). GLUT-2 locates on the base lateral membrane. It transports glucose in enterocytes into the circulation. When the gut lumen is in high glucose media, it transports from the base lateral membrane into the apical membrane transiently. The last one is the uptake of glucose into the cell by osmosis. In our study, Caco2 monolayer cells were pretreated with phloridzin or phloretin to study the possible involvement of SGLT-1 or GLUT-2 in the uptake of LBP. Phloridzin and phloretin are inhibitors for SGLT-1 or GLUT-2 and they won’t get into cells or disturb the intracellular glucose metabolism. Our results support the hypothesis that LBP may be transported across the Caco2 monolayer cells via the same transporters that transport glucose, since LBP uptake was significantly inhibited by phloridzin and also affected by phloretin. Therefore it is possible that LBP inhibits glucose absorption mainly via competition for SGLT-1. In addition, administration of LBP down regulated the expression of SGLT-1.

## 4. Materials and Methods 

### 4.1. Materials and Reagents

Fetal bovine serum (FBS) was purchased from Gibco BRL (Gaithersburg, MD, USA). Nonessential amino acids, glucose (HK) assay kit, l-rhamnose, l-fucose and dextran standard were purchased from Sigma-Aldrich Chemical Co. (St. Louis, MO, USA). d-Glucose, d-mannose, d-galactose, d-ribose and d-arabinose were purchased from Shanghai Chemical Reagent Co. (Shanghai, China). d-Xylose was purchased from Acros Organics (Geel, Belgium). Penicillin, streptomycin, trypsin and DMSO were purchased from Amresco (Solon, OH, USA). Phloridzin was purchased from the Chengdu Food and Drug Inspection Institute (Chengdu, China). All other reagents were of analytical grade. Total RNA Extractor (TRIZOL) was purchased from Sangon Biotech (Shanghai, China). RevertAid™ First-Strand cDNA Synthesis Kit and Power SYBR^®^ Green PCR Master Mix used in real-time PCR were purchased from Thermo Scientific (Waltham, MA, USA). Cell culture plates (12 mm polycarbonate membrane, 0.4 μm pore size, 1.12 cm^2^ surface area) were purchased from Corning (Corning, NY, USA). 

### 4.2. Preparation of LBP

LBP was prepared as described previously [1]. Dried *L. barbarum* was made into a powder and decocted with water (60 °C) by a traditional method used for Chinese medicinal herbs after degreasing. Then it was filtered by regenerated cellulose membranes of 300 kDa, 100 kDa, 80 kDa, 50 kDa and 30 kDa (0.2 MPa, 60 °C) after centrifuging. The resulting fraction was retained and vacuum-dried at 40 °C. Neutral sugars were determined by phenol-H_2_SO_4_ [12], acidic sugars by carbazole [13] and proteins by the Coomassie Brilliant Blue G-250 method [14].

### 4.3. Characterization of LBP

#### 4.3.1. Analysis of Monosaccharide Composition

The monosaccharide composition of LBP was carried out by GC/MS (GCMS-QP2010 Ultra, Kyoto, Japan) on an Rxi-5Sil ms column (0.25 μm × 0.25 mm × 30 m, Agilent, Santa Clara, CA, USA). LBP was dissolved with pyridines at 75 °C for 0.5 h and vibrated every 10 min [15]. Sample was diluted with 0.2 mL hexamethyldisilazane (HMDS) and trifluoroacetic acid (TFA) for 2 min after cooling down to room temperature. The chromatographic conditions used were: injection volume of sample, 1 μL; carrier gas, helium; flow rate, 1 mL/min; temperature of injector and detector, 230 °C and 260 °C; column temperature programmed from 140 °C to 198 °C at 2 °C /min, holding for 4min at 198 °C, then increasing to 214 °C at 4 °C /min and 217 °C at 1 °C/min, and then holding for 4min at 217 °C; increasing to 250 °C at 3 °C/min and finally holding for 5 min at 250 °C. Eight standard monosaccharides (d-mannose, l-rhamnose, d-glucose, d-galactose, d-xylose, d-arabinose, d-ribose, and l-fucose) were used as references.

#### 4.3.2. Molecular Weight Determination 

The molecular weight of LBP was determined using an Agilent High Performance Liquid Chromatograph (HPLC) instrument equipped with a Shodex OHpak SB-802HQ (8.0 mm × 300 mm, Showa Denko, Japan) column. The sample solution was eluted with 0.71% NaSO_4_, containing 0.02% NaN_2_ solution at a flow rate of 0.5 mL/min and detected by a differential refractive detector. The molecular weight of LBP was estimated with dextran standards of known molecular weight (10 kDa, 30 kDa).

#### 4.3.3. UV and Infrared Spectral Analysis

LBP was dissolved and diluted to a proper concentration, and scanned from 200 nm to 550 nm with a UV spectrophotometer (2910 UV-vis, Hitachi, Tokyo, Japan). The infrared spectral analysis of LBP, as KBr pellets, was determined using a Fourier transform infrared spectrophotometer (IR Affinity-1, Shimadzu, Kyoto, Japan) in a frequency range of 4000–400 cm^−1^.

### 4.4. Cell Culture and Cell Confluences

Human Caco2 cells were provided by the Cell Bank of the Shanghai Institute of Cell Biology, Chinese Academy of Sciences (Shanghai, China) and maintained in α-MEM containing 10% FBS, 1% nonessential amino acids, 100 U/mL penicillin and 100 g/mL streptomycin at 37 °C in a humidified atmosphere with 5% CO_2_. The cells were subcultured every 2–4 days using a 0.125% EDTA trypsin solution. Cells from passages 5–24 were used in this experiment. Cell culture medium was changed every 2 days. The Caco2 cells were trypsinized and seeded in 12-well transwells (0.4 μm) fitted in bicameral chambers at 1.25 × 10^5^ cells/cm^2^ and were allowed to reach confluence. At 14 days–16 days post seeding (90%–100% confluence), the integrity of the monolayer was tested by phosphate buffer saline (PBS) containing phenol red to the apical chamber. After 1 h incubation at 37 °C the optical density (558 nm) of the basal well contents was measured to detect any leakage of the phenol red through the intercellular spaces. The transepithelial electrical resistance (TEER) across the cell monolayer was measured using a Millicell ERS (Millipore, Eschborn, Germany) and a value above 500 Ω × cm^2^ [16] was considered sufficient to study ingestion.

### 4.5. Uptake of LBP

Epithelial uptake of LBP was detected in triplicate. LBP at various loading concentrations (100, 200, 400 μg/mL) was suspended in the transport buffer by sonication for 30 s (Sonic Dismembrator, Fisher Scientific, New York, NY, USA). Caco2 cells grown on the membrane inserts were first rinsed with 1.5 mL of PBS and then bathed in 0.5 mL transport buffer containing LBP. A total of 1.5 mL transport buffer was added into the basal chamber. The system was incubated at 37 °C for 2 h and samples were taken from the basal chamber at 15 min, 30 min and 1 h, 2 h. The basal chamber buffer was replenished with transport buffer at each time point. Cumulative transports were the sum of the amount transported from all time points. Samples were stored at −20 °C until analysis. The total polysaccharide of samples was determined using phenol-H_2_SO_4_ [12]. Glucose in samples was also measured with a Glucose Assay Kit (Jiancheng Bioengineering Institute, Nanjing, China).

### 4.6. Uptake of LBP on Glucose Consumption Assay

Cells grown on the membrane inserts until confluence were first rinsed with 1.5 mL of PBS and then bathed separately in 0.5 mL transport buffer containing LBP (100, 200, and 400 μg/mL) + glucose (10 mmol/L), or 0.5 mL transport buffer containing glucose (5, 10 and 15 mmol/L) + LBP(200 μg/mL). A total of 1.5 mL transport buffer was added to the basal chamber. All experiments were carried out in three replicates. The system was incubated at 37 °C for 2 h and samples were taken from the basal chamber at 15 min, 30 min and 1 h, 2 h. Samples were stored at −20 °C until analysis. The glucose of samples was determined using the Glucose (HK) Assay Kit.

### 4.7. Effects of SGLT-1 or GLUT-2 during the Progress of LBP Uptake

#### 4.7.1. SGLT-1 and GLUT-2 Inhibition Assay

Phloridzin and phloretin are a SGLT-1 inhibitor and a GLUT-2 inhibitor. Phloridzin (or phloretin) was suspended in the transport buffer by sonication for 30 s. Caco2 cells grown on membrane inserts were first rinsed with PBS and then bathed in 0.5 mL phloridzin (2 mmol/L) or phloretin (2 mmol/L), 37 °C, 20 min before treatment. Then the apical solution was replaced with 0.5 mL transport buffer containing LBP (100, 200, and 400 μg/mL). A total of 1.5 mL transport buffer was added to the basal chamber. The system was incubated at 37 °C for 2 h. Samples were taken from the basal chamber at 15 min, 30 min and 1 h, 2 h. The basal chamber buffer was replenished with transport buffer at each time point. Samples were stored at −20 °C until the samples was determined. All experiments were carried out in triplicate.

#### 4.7.2. SGLT-1mRNA and GLUT-2mRNA Expression

Caco2 cells were seeded in 12-well transwells at a density of 1.25 × 10^5^/mL maintained in cultured medium containing LBP (0, 100, 200 and 400 μg/mL) and allowed to reach confluence. Total RNA from Caco2 cells was extracted using TRIZOL reagent and the cDNA was synthesized with a RevertAid™ First-Strand cDNA Synthesis Kit following the supplier's instructions. The gene mRNA expressions of SGLT-1 or GLUT-2 were determined with a Power SYBR^®^ Green PCR Master Mix. Briefly, a 25 μL PCR mixture was prepared as follows: 12.5 μL of SYBR Green PCR Master Mix (2×), 1 μL of forward primer (10 μmol/L), 1 μL of reverse primer (10 μmol/L), 1 μL of cDNA, and 9.5 μL of sterilized double-distilled water. The PCR reaction was conducted on an real-time thermal cycler system (Funglyn Biotech, Scarborough, ON, Canada) programmed as follows: GLUT-2 and GAPDH 94 °C for 5 min; 35 cycles of 94 °C for 30 s, 55 °C for 30 s, 72 °C for 30s; and 72 °C for 10 min. SGLT-1 94 °C for 5 min; 30 cycles of 94 °C for 30 s, 58 °C for 30 s, 72 °C for 60 s; and 72 °C for 10 min. The primers used were as follows: SGLT-1 (forward) 5′-TTT TGG TGG TTG TGC TGG-3′ (reverse) 5′-CTC GGA AGA TGT GGA AGG-3′ (87 bp); GLUT-2 (forward) 5′-ATT GGA ATG AGT GGG ATG-3′ (reverse) 5'-TTG CTA AAG CAG CAG GAC-3′ (94 bp); GAPDH (forward) 5′-CAC ATC GCT CAG ACA CCA-3′ (reverse) 5′-GGA TCT CGC TCC TGG AAG-3′ (258 bp). All samples were run in triplicate. The average cycle threshold (Ct) values were used for quantification using the 2^−∆∆Ct^ method.

### 4.8. Statistical Analysis

Two-way repeated measures ANOVA was used to analyze differences between groups, effects over time and their interactions, followed the GLM procedure of SPSS, version 13.0 (SPSS Inc., Chicago, IL, USA). Data at each time point were analyzed by one-way analysis of variance (ANOVA) as well as SGLT-1mRNA and GLUT-2mRNA expression. Student’s paired *t*-test was also used to compare the effect of LBP concentration. The data were presented as mean ± SD. Differences were considered significant at *p* < 0.05.

## 5. Conclusions

In this study a time-dependent effect on uptake of LBP in intestine Caco2 cells was demonstrated This finding confirmed the intestinal uptake of polysaccharides. LBP reduced absorption of glucose primarily through competitive inhibition of SGLT-1 receptor in Caco2 cells. Administration of LBP down regulated the expression of SGLT-1. These results explain the hypoglycemic activity of LBP on humans and animals. Based on the study, LBP could be an excellent auxiliary drug for glucose control because of its absence of side effects. This provides strong support for the clinical application of LBP.
